# Relationship between Serum Concentration of Uric Acid and Insulin Secretion among Adults with Type 2 Diabetes Mellitus

**DOI:** 10.1155/2011/107904

**Published:** 2011-12-14

**Authors:** J. A. Robles-Cervantes, M. G. Ramos-Zavala, M. González-Ortiz, E. Martínez-Abundis, C. Valencia-Sandoval, A. Torres-Chávez, C. Espinel-Bermúdez, N. J. Santiago-Hernández, S. O. Hernández-González

**Affiliations:** Medical Research Unit in Clinical Epidemiology, Specialties Hospital, Medical Unit of High Specialty, West National Medical Center, Mexican Institute of Social Security, Avenida Chapalita, 1300 Col. Chapalita, Guadalajara, JAL 45000, Mexico

## Abstract

To determine the relationship between serum concentrations of uric acid and insulin secretion with hyperglycaemic clamp technique among adults with type 2 diabetes mellitus (DM2) without hyperuricemia, we carried out a cross-sectional study on 45 patients of both gender. We observed correlation between uric acid with male gender *r* = 0.710 (*P* = 0.001). Also correlation between uric acid and total insulin secretion was positive *r* = 0.295 (*P* = 0.049). As well as a positive correlation adjusted for body mass index was demonstrated for the first, second, and total phases of insulin secretion, respectively, *r* = 0.438 (*P* = 0.022), *r* = 0.433 (*P* = 0.022), and *r* = 0.439 (*P* = 0.024). Serum concentration of uric acid showed a positive relationship with the total phase of insulin secretion; even in states prior to hyperuricemia, uric acid can play an important role in the function of the beta cell in patients with DM2.

## 1. Introduction

Hyperuricemia is a condition that is significantly associated with markers of metabolic syndrome such as dyslipidemia, glucose intolerance, high blood pressure, and central obesity, which are accepted as risk factors for developing cardiovascular disease. Hyperuricemia is probably associated with glucose intolerance due to various mechanisms; however, the most important is the association between insulin and renal resistance to absorption of urates [1].

Hyperuricemia has been associated with insulin resistance; however, there are few studies where the association of hyperuricemia-insulin resistance and beta cell function is evaluated. A modest positive association between concentrations of uric acid and incidence in type 2 diabetes mellitus was observed in a cohort of a Chinese population [2]. It was reported recently in another cohort that uric acid is a risk factor for development of DM2 [3]. At the same time, another study demonstrated that serum uric acid values may be useful as predictors of DM2 in adults who are glucose intolerant [4]. Cohort studies support the fact that uric acid is a risk factor for developing DM2; in a meta-analysis by Kodama et al. [5], the authors concluded that the variability of the results and control of confounding variables should be considered in the final analysis of competitive models for interpreting the results regarding the role of uric acid as a risk factor for developing DM2. In a study that reported beta cell function with HOMA in subjects with hyperuricemia, failure of beta cells to compensate for the variation in insulin sensitivity was demonstrated [6]. These studies from different population samples established an association between serum uric acid and prevalence of DM2, however a temporal effect between uric acid and different phases of insulin secretion cannot be shown by these data. The role of uric acid as a continuous variable and its relationship to insulin secretion without hyperuricemia in patients with DM2 in a model such as the clamp has not been evaluated.

The aim of this study was to determine the relationship between serum concentrations of uric acid with insulin secretion in adults with DM2 without hyperuricemia using the hyperglycaemic clamp technique.

## 2. Material and Methods

A cross-sectional analytical study was carried out in 45 subjects of both genders. Subjects were between 40 and 60 years of age and were classified as overweight or grade I obesity and diagnosis of DM2 <5 years of evolution according to the American Diabetes Association (ADA) criteria. All individuals were nonsmokers. Their body weight was stable for at least 3 months before the study. Blood pressure was less than 130/80 mm Hg. Subjects denied use of any medications known to affect metabolism. 

At the time of the study, patients were not taking hypoglycemic agents approved for glucose control or they were unaware as to the effects of uric acid. The study protocol was reviewed and approved by the hospital-based ethics committee, and written informed consent was obtained from all volunteers. Subjects were selected from metropolitan Guadalajara, Jalisco, Mexico living in the same residential area and of similar socioeconomic status.

The study was performed at 8:00 AM after a 10–12 hour overnight fast. For all participants, a clinical history was done using the following determinations (weight, height, body mass index (BMI), waist circumference (WC), hip circumference (HC), waist/hip ratio (WHR), and blood pressure (BP)) followed by laboratory tests to determine glucose, HbA1c, creatinine, uric acid, total cholesterol (TC), triglycerides (TG), very-low-density lipoprotein cholesterol (VLDL-C), high-density lipoprotein cholesterol (HDL-C), low-density lipoprotein cholesterol (LDL-C), and serum insulin concentrations. We finally determined the phases of insulin secretion using hyperglycaemic clamp technique.

Serum glucose was determined using the glucose-oxidase technique (Boehringer Mannheim GmbH, Mannheim, Germany), with an intra- and interassay coefficient of variation <3%. For determination of HbA1c levels, ion-exchange high-performance liquid chromatography was carried out (Bio-Rad Laboratories, Hercules, Calif) with an intra-assay coefficient of variation of 2.8% and 3.5% and intera-ssay coefficient of variation <3.0%. Creatinine, uric acid, and lipid profile (TC, HDL-C, TG, LDL-C, and VLD-CL) were measured enzymatically (Ortho-Clinical Diagnostics, Johnson & Johnson Company, Rochester, NY, USA) with an intra- and interassay coefficient of variation <2%. Insulin concentrations were measured using the microparticle enzymatic immunoassay method (Abbott Diagnostics Division, Japan Co. Ltd.) with an intra- and interassay coefficient of variation of 3.3 and 3.8%, respectively.

To determine the phases of insulin secretion, hyperglycemic clamp technique was performed. Briefly, two venous accesses were installed: the first was retrograde over hand veins through a 19-gauge butterfly catheter used for sample taking during the test. The hand was wrapped in a thermal pillow to achieve a local temperature of 40°C in order to arterialize the blood. The second access was installed on the contralateral arm with a 19-gauge catheter. A 20% dextrose infusion was initiated: a priming dose for 14 min equivalent to 240 mg/kg body weight followed by a maintenance dose based on body weight, basal glucose, and the glucose required throughout the test (6.9 mmol/L above basal value). At 2, 4, 6, 8, and 10 min, 5 mL of blood was taken and, after that, each 10 min for 120 min for insulin determination. At 5 min intervals, an additional 1.5 mL blood sample was taken for glucose determination to calculate the estimate of glucose metabolism as well as to adjust the rate of dextrose infusion. At the end of the test (120 min), dextrose infusion was maintained for 30 min as a precaution to avoid hypoglycemia. With the above-mentioned results and using a calculator program, first (0–10 min) and late (10–120 min) phases of insulin secretion as well as total insulin concentration (0–120 min) were calculated. Area under the curve was determined using integration of polynomials for glucose regressions and phases of insulin secretion

### 2.1. Statistical Analysis

Results were converted to the International System of Units and presented as mean ± standard deviation (SD). Pearson bivariate correlations between uric acid and clinical and laboratory variables to establish a relationship with the gender Spearman correlation were performed. The relationship of uric acid with insulin secretion and other adjusted variables was determined with the Pearson correlation test. Statistical analyses were performed using STATA/SE v.8.0 for Windows.

## 3. Results

Forty-five patients were recruited into the study (Table 1). There were 21 (46.6%) males and 24 (53.4%) females. There was no statistical significance in age according to gender (48 ± 4 versus 48 ± 5 years, *P* = 0.850) or in BMI (29.8 ± 2.7 versus 30.6 ± 3.0 kg/m^2^, *P* = 0.419) between males and females, respectively.

Males had a larger WC as well as higher BP. A positive relationship was observed for male gender, BMI, WC, and fasting insulin according to the bivariate correlation (Table 2).

We observed a positive correlation between uric acid and total insulin secretion *r* = 0.295  (*P* = 0.049) (Table 3). Also a positive correlation adjusted for BMI was demonstrated for the first and second phases, respectively, *r* = 0.438 (*P* = 0.022), *r* = 0.433 (*P* = 0.022) as well as in total insulin secretion *r* = 0.439 (*P* = 0.024) (Table 4); variables that were found significant in the bivariate model with uric acid were not significant in correlation with the phases of secretion.

## 4. Discussion

The association of hyperuricemia and development of DM2 have been observed by various investigators. However, there are no previous studies showing the possible relationship of serum uric acid with different phases of insulin secretion using a hyperglycemic-hyperinsulinemic clamp technique in subjects with DM2 without hyperuricemia. The mechanisms by which uric acid is involved in beta cell function or glucose concentrations and even the development of DM2 in the long term are uncertain. It is accepted that the most important mechanism may be that of the association of insulin resistance on renal absorption of urates [1]. Likewise, inhibition of uricase in a rat model revealed a decrease in serum insulin and hyperglycemia and rapid removal of basal insulin in *in vitro* secretion as well as reversing the effect of the removal of uric acid, suggesting an interference with the mechanism of insulin secretion [7]. Another study reported evidence of the involvement and role of uric acid on the alteration of the primary function of the beta cell and suggests the presence of an arginine residue combined with a critical site of the cell. The default is probably due more to an alteration in the secretion from the stimulus with a secretagogue than to an adverse effect on the viability of the beta cell [8]. Recent studies have reported the pro-oxidative capacity of uric acid in the differentiation of preadipocytes to adipocytes, an increase in reactive oxygen species (ROS) by activation of the NADPH oxidase, and sustained inflammation, mechanisms that may promote insulin resistance and impaired insulin secretion [9].

Another study explored the relationship between beta cell function and uric acid. Insulin secretion was stimulated with L-arginine, and it was observed that in subjects with hyperuricemia and insulin resistance beta cell function increased from its compensatory state [10]. In a study comparing four study groups (control, type 2 diabetes: with and without obesity and type 1 diabetes), C-peptide levels were increased in patients who had type 2 diabetes and obesity. It appears that uric acid behavior is closely related with beta cell function [11]. This apparently was related to the previously mentioned information concerning residual arginine in the beta cell as well as reporting on the stimulation of insulin secretion in pancreatic tissue of rat where it was observed that uric acid has no influence on insulin secretion when stimulated to euglycemic concentrations (100 mg/100 mL). However, when insulin secretion was stimulated to hyperglycemic concentrations (300 mg/100 mL), insulin secretion was increased by the addition of uric acid >100% [12]. In the present study, hyperglycemic clamp (200 mg/100 mL) technique was used to stimulate insulin secretion, and a positive relationship was demonstrated between the serum concentration of uric acid and the total phase of insulin secretion. The first phase of secretion was the most important one and it influenced in an important way on the positive correlation; this was probably caused by a compensatory response of the beta cell in subjects with DM2 without hyperuricemia.

## 5. Conclusions

Serum concentration of uric acid showed a positive relationship with the total phase of insulin secretion; even in states prior to hyperuricemia, uric acid can play an important role in the function of the beta cell in patients with DM2.

## Figures and Tables

**Table 1:** Clinical and biochemical characteristics in study.

Variables	Total *n* = 45
Age, years	48.7 ± 4.9
Body mass index, kg/m^2^	
Male	29.8 ± 2.7
Female	30.6 ± 3.0
Waist circumference, cm	
Male	106.0 ± 11.4
Female	97.7 ± 8.6
Systolic blood pressure, mmHg	117.1 ± 9.4
Diastolic blood pressure, mmHg	76.7 ± 6.9
Glucose, mg/dL	138.7 ± 29.0
HbA1c, %	7.7 ± 1.0
Uric acid, mg/dL	5.2 ± 1.0
Cholesterol, mg/dL	181.9 ± 30.4
Triglycerides, mg/dL	182.4 ± 68.5
LDL-C, mg/dL	106.1 ± 25.6
HDL-C, mg/dL	37.3 ± 10.5
Insulin, *μ*U/mL	14.7 ± 7.2
Glucose metabolized*, mg/kg/min	3.6 ± 0.7
Phase 1 of Insulin secretion, *μ*U/mL	18.2 ± 11.5
Phase 2 of Insulin secretion, *μ*U/mL	37.5 ± 21.3
Insulin secretion total, *μ*U/mL	29.8 ± 16.4

LDL-C: low-density lipoprotein cholesterol; HDL-C: high-density lipoprotein cholesterol.

*Calculated with the clamp technique.

**Table 2:** Bivariate correlation between uric acid and variables of the study.

	*rn* = 45	*P*	95% CI
Male*	0.710	0.001	0.000–0.025
BMI, kg/m^2^	0.381	0.009	0.087–0.603
WC, cm	0.468	0.001	0.038–0143
SBP, mmHg	−0.127	0.408	−0.364–0.151
DBP, mmHg	−0.057	0.708	−0.920–0.631
Glucose, mg/dL	−0.013	0.931	−0.836–0.767
HbA1c, %	0.078	0.607	−2.666–4508
Cholesterol, mg/dL	0.106	0.484	−0.061–0.126
TG, mg/dL	0.247	0.101	−0.002–0.032
LDL-C, mg/dL	0.053	0.727	−0.184–0.263
HDL-C, mg/dL	−0.185	0.222	−0.248–0.059
Insulin, *μ*U/mL	0.331	0.026	0.017–0.259
M, mg/kg/min	−0.192	0.204	−3.296–0.725

BMI: body mass index; WC: waist circumference; SBP: systolic blood pressure; DBP: diastolic blood pressure; TG: triglycerides; LDL-C: low-density lipoprotein cholesterol; HDL-C: high-density lipoprotein cholesterol; M: glucose metabolized (calculated with the clamp technique).

*Spearman correlation.

**Table 3:** Correlation between uric acid and insulin secretion phases.

	Uric acid		
	*r*	*P*	95% CI
Phase 1 of ScI, *μ*U/mL	0.291	0.052	−0.000–0.175
Phase 2 of ScI, *μ*U/mL	0.279	0.063	−0.002–0.097
ScI total, *μ*U/mL	0.295	0.049	0.037–0.122

95% CI: 95% confidence interval; ScI: insulin secretion.

**Table 4:** Correlation between uric acid and insulin secretion phases adjusted by body mass index.

	Uric acid		
	*r*	*P*	95% CI
Phase 1 of ScI, *μ*U/mL	0.438	0.022	0.039–0.488
Phase 2 of ScI, *μ*U/mL	0.433	0.022	0.041–0.498
ScI total, *μ*U/mL	0.439	0.024	0.036–0.486

95% CI: 95% confidence interval; ScI: insulin secretion.
